# Knowledge, attitudes, and practices of nurses in preventing unplanned extubation of orotracheal intubation in the adult intensive care unit: a multicenter questionnaire survey

**DOI:** 10.3389/fmed.2026.1807204

**Published:** 2026-04-10

**Authors:** Yanping Lu, Qin Ma, Xiaolin Sun, Furong Li, Shiqin Pan

**Affiliations:** Qinghai Provincial People’s Hospital, Xining, Qinghai, China

**Keywords:** beliefs, knowledge, practices, prevention, unplanned extubation of tracheal intubation

## Abstract

**Background:**

Endotracheal intubation is a common procedure performed in the intensive care unit (ICU), which can lead to life-threatening outcomes.

**Aim:**

To understand the knowledge, attitudes, and practices, as well as influence factors, on nurses for unplanned extubation prevention in patients with orotracheal intubation in intensive care unit.

**Study design:**

A total of 698 ICU nurses from 26 tertiary hospitals were enrolled in January 2022. Their knowledge, attitudes, and practices (KAP) of unplanned extubation prevention were collected.

**Results:**

The KAP scores were 3.31 ± 1.50, 166.94 ± 23.83, and 126.88 ± 16.43, respectively, and the scoring rates for these dimensions were 33.07, 90.24, and 87.50%, respectively. The standard score for knowledge was 33.07 ± 15.03. Multivariate linear regression analysis revealed that while years of experience and region were statistically significant factors, they explained only a small proportion of the variance in KAP scores, suggesting that other unmeasured factors also play an important role. Nurses with ≤5 years of clinical experience had poorer knowledge than those with >5 years of clinical experience. Nurses at hospitals located in the plateau region had poorer KAP regarding unplanned extubation than those in the plain region. Albeit with regional and experience-dependent variations, most nurses had positive attitudes for unplanned extubation prevention, but their knowledge and practices require improvements.

**Conclusion:**

The survey indicated that the attitude, and practice levels regarding unplanned orotracheal extubation among ICU nurses were moderate, suggesting significant room for improvement. Notably, knowledge scores were relatively low, particularly among nurses in plateau regions, highlighting the necessity of prioritizing training in this area.

## Introduction

Tracheal intubation is one of the most commonly performed procedures to supply oxygen, maintain ventilation, and protect the airway in critically ill patients (1, 2). Unplanned extubation (UEX) describes the removal of a tracheal tube by a patient without achieving the extubation endpoint or the dislodgement of a tracheal tube because of improper operation by the medical staff or accidental slippage (3–5). UEX can lead to potentially life-threatening outcomes, such as asphyxia, arrhythmia, bronchospasm, and aspiration pneumonia (6, 7). In the intensive care unit (ICU), the reported incidence of UEX ranges 3.0–22.1%. In particular, self-extubation by patients accounts for up to 95.1% of all cases of UEX. The risk of UEX in patients with orotracheal intubation was 2.13-fold higher than that in patients with nasal tracheal intubation (8–10). Preventing UEX is an important responsibility of the nursing staff to ensure that high-quality healthcare is delivered to patients in the ICU (10).

The Knowledge, Attitudes, and Practices (KAP) model, posits that knowledge is the foundation for forming positive attitudes and beliefs, which in turn lead to the adoption of healthy practices (11, 12). This theoretical framework is widely used in health education and behavior change research (13). In the context of patient safety, adequate knowledge about risk factors and prevention strategies fosters a proactive attitude among healthcare providers, which is a critical precursor to the consistent and correct execution of evidence-based clinical practices (14). Therefore, understanding the KAP of ICU nurses regarding UEX prevention is essential for identifying gaps and designing effective interventions to enhance patient safety (15–17). In UEX prevention, nurses’ attitudes toward UEX can directly affect their nursing practices, which in turn affects patients’ outcomes (18–20). However, there are few systemic nursing studies on the KAP model for UEX prevention.

Previously, we developed and validated a questionnaire to evaluate knowledge, attitudes, and practices for UEX prevention (21). In the current study, we applied this questionnaire to investigate the KAP of ICU nurses concerning UEX prevention. We also analyzed the factors influencing their KAP with the aim of improving ICU clinical nursing practice to reduce the incidence of UEX.

## Materials and methods

### Study design

We performed a multicenter cross-sectional study of ICU nurses from 26 tertiary hospitals in China. The study protocol was approved by the ethics committee of Qinghai Provincial People’s Hospital (Ethical No. 2023-042). All study participants provided written informed consent.

### Participant selection

ICU nurses were screened and enrolled in the study by a convenience sampling method. The inclusion criteria were a current state-registered nursing license and engagement in clinical critical care nursing care for ≥1 year. Nurses with no responsibility for direct patient care for more than 3 months were excluded from the study.

### Sample size

The sample size was determined based on the requirements for multivariate analysis (22), which recommend that the sample size should be at least 10 to 15 times the number of independent variables. In this study, there were 8 independent variables. Considering a 10% rate of invalid samples, the required sample size was at least 90 to 123 cases. Ultimately, 698 nurses were included in this study.

### Questionnaire survey

During this cross-sectional study, the questionnaire survey was submitted to eligible nurses. The survey assessed nurses’ general information and their knowledge, attitudes, and practices concerning UEX prevention.

The general information collected included the hospital name, area, age, sex, nationality, ICU specialization (yes or no), educational background, professional title, and years of clinical experience as a nurse.

The questionnaire on the knowledge, attitude, and practices model for UEX prevention in patients in the ICU with orotracheal intubation was previously developed and published by our team (21). Briefly, a questionnaire item pool was formed by searching a large number of studies, and the questionnaire assessed risk factors, prevention strategies, and relevant knowledge, attitudes, and practices related to UEX combined with the theoretical framework of the knowledge, attitude, and practices model. After two rounds of Delphi expert consultation, pre-experimentation, and item analysis, the questionnaire items were screened. The final questionnaire consisted of three dimensions and 76 items. The knowledge dimension had 10 items and a total of 10 points. The knowledge standard score was calculated as follows: (total average score/total score) × 100. The attitude dimension had 37 items, and it was scored using a 5-point Likert scale as follows: 1, “strongly disagree;” 2, “disagree;” 3, “uncertain;” 4, “agree;” and 5, “strongly agree.” The total score ranged 37–185 points. A higher score suggested a more positive attitude. The practice dimension had 29 items, and it was scored using a 5-point Likert scale as follows: 1, “never;” 2, “occasionally;” 3, “sometimes;” 4, “often;” and 5, “always.” The total score ranged 29–145 points. A higher score suggested better clinical practice behaviors. The scoring rate for each item was calculated as the number of correct answers divided by the total number of participants. The total score of the entire questionnaire ranged 76–340. Cronbach’s *α* of the questionnaire was 0.981. Cronbach’s α values for the knowledge, attitude, and practice dimensions were 0.966, 0.996, and 0.981, respectively. The test–retest reliability was 0.843, and the total content validity was 0.96. Exploratory factor analysis demonstrated that the cumulative contribution rate of variance was 85.52%, indicating satisfactory reliability and validity.

### Data collection

An electronic questionnaire was used to collect data. First, we explained the purpose of the survey and the principle of anonymity with the head nurse and every potential participating nurse in the unit. Second, an electronic questionnaire with an informed consent was sent to all potential participating nurses and invited them to fill it out via their mobile phones. Answers were required to all questions. Each question could only be answered once.

### Statistical analysis

The sample size was determined according to the basic standard of 5–10 times the number of questionnaire items. As the questionnaire featured 76 items, at least 380 valid questionnaires were required for this analysis.

All data were analyzed using SPSS 23.0 (IBM, Armonk, NY, United States). Continuous data were presented as the mean ± standard deviation and compared using the independent-samples *t*-test or one-way ANOVA. Categorical data were presented as numbers with percentages and compared using the chi-squared or Fisher’s exact test. Two-tailed *p* < 0.05 was considered statistically significant.

## Results

### General information on participants and questionnaires

In total, 713 questionnaires were collected, 698 of which were valid (effective recovery rate of 97.89%), and the cohort included 124 men (17.77%) and 574 women (82.23%). The mean participant age was 30.18 ± 4.78 years, and the average duration of clinical experience as a nurse was 6.54 ± 3.97 years.

### Scores for the knowledge, attitudes, and practices dimensions

The total score of the questionnaire was 297.13 ± 35.38, and the scoring rate was 87.39%. The knowledge dimension score was 3.31 ± 1.50 points, and the standard score and scoring rate were 33.07 ± 15.03 points and 33.07%, respectively. The attitudes dimension score was 166.94 ± 23.83, and the scoring rate was 90.24%. The practice dimension score was 126.88 ± 16.43, and the scoring rate was 87.50%. The scores of the attitudes and practices dimensions were ranked. The top and bottom five items are presented in Table 1.

**Table 1:** The scores for the top five and bottom five ranked items in the attitudes and practices dimensions (*N* = 698).

Dimension and item	Score
Attitudes	166.94 ± 23.83
Top 5 items	
A18: Should proactively learn and master knowledge related to ICU delirium	4.55 ± 0.64
A27: Should use appropriate physical restraint assessment tools	4.54 ± 0.66
A23: Should choose appropriate oral intubation fixation materials and methods based on the patient’s consciousness level, compliance, and skin condition	4.54 ± 0.67
A28: Should develop a restraint observation record form and maintain nursing records	4.54 ± 0.67
A31: It is very necessary to timely assess the effects of analgesia and sedation	4.54 ± 0.68
Bottom 5 items	
A32: In the absence of contraindications, for patients with RASS scores ≤ − 2, spontaneous awakening trials and spontaneous breathing trials should be conducted daily	4.48 ± 0.72
A1: Should timely understand and meet the needs of patients who are orally intubated and alleviate adverse stimuli	4.47 ± 0.76
A2: Should form a quality control team to prevent unplanned extubation in orally intubated patients	4.46 ± 0.77
A3: If patients have been mechanically ventilated for more than 24 h, their extubation conditions should be assessed daily	4.46 ± 0.77
A33: Should avoid excessive sedation of patients and reserve sedatives only for special cases/indications (e.g., elevated intracranial pressure)	4.43 ± 0.78
Practices	126.88 ± 16.43
Top 5 items	
P10: Change the fixation tape and oral pad for orally intubated patients in a timely manner	4.51 ± 0.62
P9: Measure and record the length of the oral intubation before and after oral care	4.50 ± 0.61
P8: Assess the fixation status of the oral intubation before and after airway care	4.49 ± 0.60
P15: Provide informed consent regarding restraints to patients/families and explain the importance of physical restraints	4.49 ± 0.60
P19: Regularly assess the effects of analgesia and sedation, and assess whenever there are significant changes in the patient’s condition or restlessness	4.45 ± 0.62
Bottom 5 items	
P21: Correctly use delirium assessment scales	4.25 ± 0.90
P25: Conduct early mobilization-related theory and skills training	4.22 ± 0.79
P2: Actively learn knowledge related to preventing unplanned extubation in orally intubated patients	4.19 ± 0.76
P3: Organize/actively participate in training related to preventing unplanned extubation in orally intubated patients	4.16 ± 0.77
P1: Organize/actively participate in quality control for preventing unplanned extubation in orally intubated patients	4.11 ± 0.84

Data are presented as the mean ± standard deviation. The analysis is based on the full sample of 698 nurses.

### Associations of knowledge, attitudes, and practices with the nurses’ characteristics

The comparison of scores for knowledge, attitudes, and practices regarding UEX prevention according to nurses’ characteristics revealed no significant differences concerning sex, ethnicity, education level, and professional title (*p* > 0.05). However, significant differences were identified for knowledge scores according to whether the nurse was an ICU specialist, the number of years of clinical experience, and age (*p* < 0.05). Additionally, significant differences in attitudes were identified by region and age (*p* < 0.05). Differences in practices were also statistically significant according to the region, years of experience, and age (*p* < 0.05). Detailed findings are listed in Table 2.

**Table 2:** Comparisons of knowledge, attitudes, and practices scores for unplanned extubation prevention based on nurses’ characteristics (*N* = 698).

Characteristics		*n*	(%)	Knowledge	Attitudes	Practices	Total score
Sex	Male	124	17.77	3.11 ± 1.49	168.81 ± 25.58	128.61 ± 18.78	300.53 ± 39.78
	Female	574	82.23	3.35 ± 1.50	166.54 ± 23.47	126.51 ± 15.89	296.39 ± 34.38
*t*				−1.119	0.68	0.916	0.836
*p*				0.264	0.497	0.36	0.404
ICU specialty nurse	No	372	53.30	3.14 ± 1.47	168.88 ± 21.11	126.56 ± 17.08	298.59 ± 34.52
	Yes	326	46.70	3.5 ± 1.52	164.72 ± 26.50	127.24 ± 15.69	295.46 ± 36.36
*t*				−2.227	1.63	−0.382	0.823
*p*				0.027	0.104	0.702	0.411
Ethnicity	Han	608	87.11	3.33 ± 1.49	166.55 ± 24.51	127.28 ± 16.51	297.16 ± 36.02
	Minority	90	12.89	3.13 ± 1.58	169.58 ± 18.61	124.16 ± 15.75	296.87 ± 31.00
*t*				0.828	−0.795	1.193	0.053
*p*				0.408	0.427	0.234	0.958
Education	Associate’s degree	132	18.91	3.2 ± 1.32	165.24 ± 20.72	126.39 ± 17.53	294.83 ± 35.46
	Bachelor’s degree or higher	566	81.09	3.33 ± 1.54	167.34 ± 24.52	126.99 ± 16.19	297.66 ± 35.40
*t*				−0.657	−0.642	−0.266	−0.584
*p*				0.511	0.521	0.79	0.559
Region	Plain	296	42.41	3.4 ± 1.74	170.50 ± 25.40	130.58 ± 16.27	304.48 ± 35.48
	Plateau	402	57.59	3.24 ± 1.31	164.32 ± 22.31	124.15 ± 16.05	291.71 ± 34.39
*t*				0.982	2.411	3.676	3.382
*p*				0.327	0.016	0	0.001
Title	Junior	546	78.22	3.28 ± 1.50	166.40 ± 23.99	126.89 ± 17.00	296.57 ± 36.16
	Intermediate	136	19.48	3.38 ± 1.57	168.59 ± 24.02	126.51 ± 14.77	298.49 ± 33.74
	Senior	16	2.29	3.63 ± 1.19	171.50 ± 17.03	129.50 ± 9.52	304.63 ± 20.40
*F*				78.22	0.313	0.379	0.118
*p*				19.48	0.732	0.685	0.889
Years of experience	≤5 years	292	41.83	3.08 ± 1.54	168.73 ± 21.87	129.62 ± 16.93	301.43 ± 34.42
	>5 years	406	58.17	3.47 ± 1.46	165.65 ± 25.13	124.91 ± 15.81	294.03 ± 35.82
*t*				−2.455	1.193	2.669	1.936
*p*				0.015	0.234	0.008	0.054
Age group	20–25 years	96	13.75	2.75 ± 1.33	172.85 ± 18.35	130.88 ± 18.03	306.48 ± 32.73
	26–30 years	312	44.7	3.44 ± 1.54	168.16 ± 23.57	128.16 ± 17.54	299.76 ± 35.76
	31–35 years	204	29.23	3.19 ± 1.45	161.37 ± 27.81	123.9 ± 14.46	288.46 ± 37.95
	36–40 years	86	12.32	3.74 ± 1.53	169.12 ± 17.15	124.84 ± 13.57	297.7 ± 26.10
*t*				4.12	3.154	2.638	3.525
*p*				0.007	0.025	0.05	0.015

ICU, intensive care unit. Data are presented as the mean ± standard deviation.

### Influence of the nurses’ characteristics on their knowledge, attitudes, and practices

Multivariate linear regression analysis was performed using the knowledge, attitudes, and practices of nurses for UEX prevention as dependent variables and the characteristics of nurses identified in univariate analysis as the independent variables (Table 3). The variable *region* was statistically associated with the total survey score and each individual dimension (knowledge, attitudes, and practices). In addition, the variable years of experience was also associated with knowledge. However, the regression models exhibited low explanatory power, with adjusted *R*^2^ values ranging from 0.014 to 0.035. This indicates that while years of experience and region are significant factors, they collectively explain only a small fraction of the variance in KAP scores. This suggests that other unmeasured variables, such as hospital safety culture, quality of training programs, or individual psychological factors, likely play a substantial role in shaping nurses’ KAP regarding UEX prevention.

**Table 3:** Multivariate linear regression analysis for nurse characteristics associated with knowledge, attitudes, and practices for unplanned extubation prevention (*N* = 698).

Questionnaire dimensions	Variable	Unstandardized coefficients		Standardized coefficients	*t*	*p*
		*β*	Standard error			
Knowledge	Constant	3.131	0.816		3.837	<0.001
	Years of experience	0.066	0.031	0.174	2.113	0.035
	Region	−0.343	0.177	0.113	−1.94	0.053
Attitudes	Constant	176.682	4.234		41.725	<0.001
	Region	−6.182	2.564	−0.128	−2.411	0.016
Practices	Constant	137.008	2.887		47.451	<0.001
	Region	−6.427	1.748	−0.194	−3.676	<0.001
Total score	Constant	317.248	6.236		50.877	<0.001
	Region	−12.768	3.775	−0.179	−3.382	0.001

Knowledge: *r*^2^ = 0.047, adjusted *r*^2^ = 0.025, *F* = 2.111, *p* = 0.034; Attitudes: *r*^2^ = 0.016, adjusted *r*^2^ = 0.014, *F* = 5.813, *p* = 0.016; Practices: *r*^2^ = 0.037, adjusted *r*^2^ = 0.035, *F* = 13.514, *p* < 0.001; Total score of the questionnaire: *r*^2^ = 0.032, adjusted *r*^2^ = 0.029, *F* = 11.437, *p* = 0.001. Years of experience: ≤5 years = 1, >5 years = 2; Region: plain = 1, plateau = 2.

### Percentage of correct answers to each item in knowledge dimension

The knowledge questionnaire included 10 items in the knowledge questionnaire, and the correct answer rate was 9.17–67.34% (Table 4). The item with the lowest correct answer rate was “The time to evaluate the constraint demand.” The item with the highest correct answer rate was “The best distance from the orotracheal intubation to the incisors.”

**Table 4:** Correct answer rate for each item in the knowledge questionnaire (*N* = 698).

Entry	Correct answer, *n* (%)
The interval for checking the tracheostomy tube cuff pressure should be at least:	86 (12.32)
The interval for assessing pain in patients with oral tracheal intubation should be:	268 (38.40)
The interval for performing oral care for patients with oral tracheal intubation should be:	320 (45.85)
The optimal distance from the oral tracheal tube to the incisors is:	470 (67.34)
The minimum distance that the patient’s hands should be from the oral tracheal tube when using upper limb restraints is:	70 (10.03)
The timing for assessing the need for restraints is:	64 (9.17)
How to select the depth of sedation for patients with oral tracheal intubation:	116 (16.62)
Which of the following measures is not recommended for preventing and treating delirium:	466 (66.76)
The preparation work that a nurse should do before implementing early mobilization in a multidisciplinary collaboration is:	252 (36.10)
Indications for pausing early mobilization include:	196 (28.08)

## Discussion

In this multicenter questionnaire survey, we assessed the KAP of ICU nurses regarding UEX prevention. Our findings reveal a significant disconnect with in the KAP model: while nurses hold predominantly positive attitudes—a crucial mediator for behavior change—their foundational knowledge (33.07%) is conspicuously inadequate. This disparity highlights that simply having a positive disposition is in sufficient to ensure optimal clinical performance, targeted knowledge enhancement is critical. This pattern is consistent with previous reports, such as that by Qian et al. (15, 19), and underscores a critical area for intervention. It suggests that nursing managers and hospitals must move beyond merely fostering good attitudes and instead focus on robust educational strategies to bridge the knowledge-to-action gap. Specifically, targeted, specialized training in UEX prevention is essential to bridge the knowledge-to-action gap and promote standardization of clinical care.

### Knowledge deficits and educational needs

The knowledge of nurses concerning UEX prevention was insufficient. Our study demonstrated that the knowledge score for nurses in tertiary hospitals was 3.31 ± 1.50 points, and the scoring and correct answer rates were 33.07 and 9.17–67.34%, respectively, suggesting an overall low knowledge level for UEX prevention. Some items, such as “The best distance between orotracheal intubation and incisors.” “Measures for prevention and treatment of delirium.” and “Oral care interval for patients with orotracheal intubation,” had high correct answer rates, probably because of the daily involvement in these clinical practices by ICU nurses. The lowest score in the knowledge dimension was for the item “The timing of assessment of restraint needs,” and the correct answer rate was only 9.17%. This might be related to the lack of formal training and learning in this area. The measurement of tracheal tube cuff pressure is an important step in the airway management of critically ill patients (11). In this survey, we found that only 12.32% of ICU nurses realized the importance of regular measurements of tracheal tube cuff pressure. In the study by Qiu et al. (16), the common causes of UEX were inadequate awareness, low responsibility, inaccurate timing of sedation, inappropriate sedation depth, and early awakening. In this study, the correct answer rate among ICU nurses concerning how to choose the sedation depth for patients with orotracheal intubation was only 16.62%. It might be difficult for nurses to evaluate and monitor the sedation level in patients with tracheal intubation, which could be related to their poor clinical experience and inadequate continuing education and learning regarding updated guidelines on sedation. Therefore, nursing managers and hospitals should pay attention to the professional development of nurses, increase the specialized learning and training opportunities available to nurses, and strengthen their knowledge related to UEX prevention.

### Attitudes: a strong foundation with notable gaps

In the current study, we found that nurses had a positive attitude for preventing UEX and realized the importance of UEX prevention in clinical work. High scores were recorded for the items “Should proactively learn and master the knowledge related to ICU delirium,” “Should use appropriate physical restraint assessment tools,” and “Should choose appropriate oral intubation fixation materials and methods according to the patient’s consciousness level, cooperation degree, and skin condition.” These measures could actively prevent UEX. However, the score for the item “Should establish a quality control team for preventing unplanned extubation of orotracheal intubation” was only 4.46 ± 0.77 points, which was relatively low. This might be related to the fact that the nurses did not realize the importance of quality control in preventing UEX. Nursing managers should establish a quality control standard and a normalized management system for UEX prevention. In addition to this item, the item “Should avoid excessive sedation of patients and reserve sedatives only for special cases/indications” also received a low score. This might be attributable to the fact that the daily evaluation of weaning conditions and the adjustment and administration of sedatives were mostly performed by physicians or respiratory therapists opposed to nurses with little experience in sedative use. The lack of awareness concerning the importance of a quality control team for UEX prevention suggested that nursing managers and hospitals should establish a management system and better quality control projects to improve the UEX awareness among nursing staff.

### Practices: the gap between attitude and action

In the practices dimension, high scores were observed for the items “Timely replacement of tracheal intubation fixed tape and tooth pad for patients with orotracheal intubation,” “Measurement and recording of the length of orotracheal intubation before and after oral care,” and “Evaluation of the fixation of orotracheal intubation before and after airway care,” indicating that most nurses could effectively manage the artificial airway during their clinical work. The scores for the items “Correct use of delirium assessment scale” and “Training of early activity-related theories and skills” were low, probably because of the lack of awareness among nursing staff that the daily assessment of delirium reduces the incidence of UEX. Liu et al. (23), mentioned that early activities could effectively reduce the incidence of delirium and minimize the risk of UEX among patients in the ICU. In addition, the score was lowest for “Organizing/actively participating in the quality control of unplanned extubation for preventing orotracheal intubation.” This might be related to the lack of a quality control system related to UEX prevention in the hospital. Therefore, hospitals should conduct training on UEX-related knowledge, regularly organize learning activities, build an educational platform, participate in quality control initiatives, and improve nurses’ awareness of safety prevention to strengthen their practices and prevent UEX.

### Factors influencing KAP: the role of experience and systemic disparities

Knowledge, attitudes, and practices can be affected by many factors. A positive attitude and continuous assessment of various risk factors are of great significance for improving knowledge, attitudes, and practices and reducing the incidence of UEX (24, 25). In the current study, we further analyzed the characteristics of ICU nurses that could influence their KAP regarding UEX prevention. Our multivariate linear regression analysis illustrated that years of clinical experience could affect UEX prevention-related knowledge. Compared with junior nurses, nurses with years greater than 5 years of experience had better knowledge of UEX prevention. An increased duration of clinical experience was associated with better knowledge of UEX prevention, consistent with the results of Silva et al. and Teng et al. (26, 27). Nurses with greater clinical experience had more opportunities for more training and education, leading to better knowledge and potentially better attitudes and practices concerning UEX prevention. Nursing managers should pay attention to the training and education of young nurses and expand the learning opportunities for junior nurses, such as strengthening communication and learning between junior and senior nurses. In addition, specialized training programs can also be provided to junior nurses to accelerate their learning and knowledge concerning UEX prevention.

In the current study, we found significant differences in attitudes and practices among nurses between the plain and plateau regions, with the score being higher for the plain region than for the plateau region. The nurses in the plateau region had insufficient knowledge and weak attitudes and practices concerning UEX prevention. This disparity likely stems from deep-seated systemic and structural factors. In China, the healthcare quality provided in the plateau region is often lower than that provided in the plain region due to challenging geographical conditions, slower economic development, and consequently, more limited medical resources and fewer professional training opportunities. The later establishment of ICUs in some plateau hospitals, as seen in this study, further compounds this issue. This finding underscores that individual nurse characteristics are only part of the picture; the broader healthcare context in which they work is a powerful determinant of their ability to acquire knowledge and implement best practices. Therefore, improving UEX prevention in under-resourced areas requires systemic interventions, such as targeted funding for professional development, the creation of tele mentoring programs, and policies to recruit and retain skilled nurses in these regions.

## Limitations

Our study had multiple limitations. The questionnaire survey was based on self-reported answers to each question, which might have resulted in social desirability bias and acquiescence bias. This is a particular concern for the “practices” dimension, as nurses may overreport their compliance with best practices. Future studies should incorporate direct observational methods or objective data from patient records to validate self-reported practices. Second, the use of convenience sampling, while practical for this multicenter study, is a non-probability method that inherently introduces selection bias. This may limit the representativeness of our sample and the generalizability of the results to the entire population of ICU nurses in China or other settings. Although we performed the survey in 26 tertiary hospital ICUs in China, the generalizability of the results to other hospital settings or ICUs in other countries required further studies. Furthermore, the low explanatory power (*R*^2^) of our regression models indicates that while we identified some significant factors, we have not captured the full complexity of influences on KAP. Future research should explore a wider range of variables, including hospital-level factors (e.g., staffing ratios, safety culture), team dynamics, and the impact of specific educational interventions. We have identified areas for improvement (such as better training, specialized education, quality control team, and resource allocation) to improve UEX prevention among ICU nurses, but further interventional studies are required to examine the efficacy of these interventions.

In conclusion, this study reveals that while ICU nurses exhibit highly positive attitudes toward UEX prevention, their knowledge remain inadequate-particularly among nurses in plateau regions and those with fewer years of experience. Therefore, interventions should prioritize strengthening knowledge through targeted training and education, especially for these vulnerable groups, to bridge the gap between positive attitudes and effective clinical practice.

## Data Availability

The original contributions presented in the study are included in the article/supplementary material, further inquiries can be directed to the corresponding author.
